# Editorial: Self-presentation during self quarantine era

**DOI:** 10.3389/fpsyg.2023.1194898

**Published:** 2023-06-13

**Authors:** Seoyeon Hong, Hyunmin Lee, Bokyung Kim

**Affiliations:** ^1^Department of Public Relations & Advertising, Ric Edelman College of Communication and Creative Arts, Rowan University, Glassboro, NJ, United States; ^2^Department of Communication, Drexel University, Philadelphia, PA, United States

**Keywords:** self-presentation, social media, pandemic (COVID-19), information seeking, social anxiety self-presentation model, Zoom fatigue, personality, self management

Over the past six decades, since Goffman (1959) proposed a very natural way of individual social interaction via *The Presentation of Self in Everyday Life*, Self-Presentation has emerged as an empirically tested perspective in how media can be managed and promoted. This promotes any behavior intended to create, modify or maintain an impression of people perceived by others. Self-presentation blooms especially with the advanced digital media because when people are uploading posts, texts, or photographs, those lead people to think of them in a particular way. This approach has been applied to why people ever want to engage in self-presentation and how they formulate impressions of themselves in the minds of other people.

In the context of digital media, media are used as tools from impression management and as self-identification in social issues. Scholars have devoted significant energies to study media effects in the changing media platforms and paid increasing attention to examine the impacts of social media and new technologies on how individuals promoted themselves in various formats. Interestingly, people are affected by self-presentational motives when they are not consciously thinking about others' impressions at the time- which makes individuals differ in the degree to which they typically manage their impressions. The current issue presents and discusses a number of critical issues in regard to how self-presentations research might address, from multiple perspectives (i.e., adult professionals, Gen Zs, and non-profits), the predictors, characteristics, mechanisms, and consequences of social media use for self-expression and self-disclosing. As a starting point, there is a growing number of research that has indicated the younger generations significantly differ from the elders in terms of technology-related self-expressing and online behaviors (Hu and Cheong, 2021). In this Research Topic of papers, Hu et al. examine the characteristics of and differences in Generation Z's social media uses and gratifications and the relationships between their online motivations, social media practices and economic capital. In this issue, Hu et al. use the Bourdieusian approach, introducing social media habitus and capital as keys to understanding the predictors and consequences of Gen Zs' social media practices (e.g., enhancing social influence and increasing social capital might play an important role in satisfying their online motivations; see Shane-Simpson et al., 2018). The survey with 221 Chinese Generation Z social media users showed that Gen Zs have different social media uses depending on two categories of online motivations: social media as communicative tools when they are doing their daily routines online; and as platforms for social capital accumulating and exchanging (SCAE) and self-expression (SE) for online socialization. The socialization (e.g., seeking help, sharing with others, developing/maintaining relationships) motivation was significantly associated with social capital accumulating and exchanging (SCAE) and self-expression (SE) on social media, regardless of their social capital. Hu et al. also found distinct social media habitus between Gen Zs from low- and upper-mid-income families. It is important to note that the richer embrace a more instrumental-rational habitus to use social media more frequently as a communicative tool; whereas the poorer value the importance of online socialization to increase their social capital, but have no more practices in related social media activities.

The connections between Gen Zs' socialization motivation, social media practices and self-expression, raises the question as to how Non-profit organizations (NPO), like individual users, communicate with other users on social media. Kim (this Research Topic) uniquely looked for the personality of NPOs' Instagram accounts, exploring the relationships between personality traits of NPO's Instagram accounts and the characteristics of the uploaded photographs at content and pixel levels. In this paper, the author focused on non-profits because they use social media as a main communication channel with the public and stakeholders (Wang and Yang, 2020); and NPOs' one-way delivery of information was the dominant motivation to use SNSs (Chung et al., 2020). This paper applied the personality framework that notes, concerning websites as online objects, websites have personalities of openness, conscientiousness, extraversion, agreeableness, and neuroticism. Kim expanded literature by examining a particular type of Instagram photographs, NPOs' Instagram accounts and their photograph features, using IBM Watson Personality Insights and an open AI. The analysis of 2,23,446 photographs on 177 Instagram accounts found that the NPOs' Instagram accounts revealed high in openness and agreeableness but low in extraversion and neuroticism. Interestingly, while openness and agreeableness were the personality traits that associated the most with the photograph features, neuroticism was associated mainly with facial features (e.g., number of faces, close-up, face ratio, age, gender and emotions), and conscientiousness with pixel color features. In addition, openness and agreeableness showed correlations in opposite directions with content category features, facial features, and visual features, suggesting that these two traits were visually manifested in an opposite manner when NPOs presenting Instagram photographs. Also, the personality traits of NPOs' Instagram accounts, except neuroticism, were predicted from the photograph features with an acceptable level of accuracy, supporting previous research neuroticism is the most difficult trait to predict. All in all, Kim suggests that social media communications and self-expression of NPOs can be understood from the perspective of personality; and that NPOs should tailor Instagram messages that correspond well with organizations' personalities, leading to a stronger influence on their communication with the public.

The applications of self-presentation have entered new domains with the explosion of virtual meetings (e.g., Zoom) that accelerated during the COVID-19 pandemic. Virtual meeting rooms, or video conferences, have been a staple for businesses and academic institutes for several decades (e.g., Sprey, 1997), however, during the pandemic it was often times the only option to conduct work, make social connections, and create some semblance of a normal social life. At the same time, with virtual meetings becoming the default mode for any given communication setting, new phenomena such as Zoom fatigue have prompted individuals to turn off cameras to reduce the intensity of excessive close-up eye contact and decrease cognitive overload (Bailenson, 2021). This has created interesting social dynamics in meetings, with differing opinions as to whether webcam use is required for good group morale and effective communication. In this issue, Zabel et al. approach webcam use by applying social norms theory. The authors argue that all considered, switching on webcams are important as the “media richness” available through audiovisuals make information interpretation more effective. The authors surveyed 393 adults in universities and workplaces. Findings from their survey revealed that for professionals, both descriptive norms (e.g., percentage of participants who had their webcams turned on in that specific meeting) and injunctive norms (e.g., other participants in the online meeting expect me to turn my webcam on) significantly predicted webcam activation in a meeting, whereas the group size was not related to webcam activation. For the university group, only the descriptive norm significantly predicted webcam activation. More interestingly, those in the university group reported untidy surroundings, being unprepared, feeling observed, and doing other things on the side as reasons for turning the camera off significantly higher than the professional group. So while webcam use is prompted by desires to comply with the social norm, saving self-image by controlling the amount of self-presentation information available via webcams seems to be a strong factor inactivating the camera: Participants turn it off when it makes undesirable information about oneself accessible to other parties.

Meanwhile, Lopez and Polletta turn their attention to social media, examining how people control their Instagram pages to evaluate their sense of self-worth and more importantly, reduce anxiety. The relationship between social media use, particularly Instagram, and mental health continues to be an important issue for scholars, as the demographic shift points to a hyperconnected society where more than 60% (Gen Z and Millennials combined) of the world population grew up with social media, and use it as a primary source for all information needs (citation needed). Research to date primarily shows negative associations between social media use and mental health measures: increased social media use was associated with depression, lower self-esteem, and warped body image especially among adolescents (e.g., Vogel et al., 2014; Tandoc et al., 2015; Cohen et al., 2017). In their study, Lopez and Polletta extend existing literature by presenting a Social Anxiety and Self-Presentation (SASP) Model, mainly. They examine social anxiety as an important predictor for self-presentation control behaviors on Instagram, such as disabling the comments function, editing photographs and videos, and editing the captions of the posts. Moreover, they argue that it is social anxiety that also strengthens the magnitude of Instagram's influence on one's self-worth evaluations. Through a survey of 247 adults, the scholars found that those with higher social anxiety scores had higher Instagram contingent self-worth, which correlated with several Instagram content control behaviors, such as spending more time editing captions as well as photographs and videos. These results held true after adjusting for participants' age, sex, and Instagram daily screen time, suggesting that social anxiety is an important factor when characterizing Instagram users' sense of self-worth (tied to the platform) and specific content control behaviors to preserve self-image and reduce anxiety.

The four articles featured in the current special call are among the first few to study self-presentation with the digital context during the COVID era. Guided by the theoretical, practical, and methodological strides made in these studies, I hope following research in this area will continue to discover and more fully explain how humans are evolved to camouflage as social media users and how communication should be practiced, both strategically and ethically.

## Author contributions

SH: developing content for articles, writing editorial piece, meeting, reviewing articles, and assigning reviewers. HL and BK: writing editorial piece, meeting, reviewing articles, and assigning reviewers. All authors contributed to the article and approved the submitted version.
